
JOURNAL INFORMATION
==============================
NLM Title Abbreviation: Curr Med Sci
Journal ID: Curr Med Sci
NLM Journal ID: 101729993

Current Medical Science

ISSN: 2096-5230
EISSN: 2523-899X

ARTICLE INFORMATION
==============================
PMCID: PMC7801311
PMID: 33351165
DOI: 10.1007/s11596-020-2278-x
Article ID: 2278
Article version: 1
Subjects: Erratum

Erratum to: Application of Virtual Reality Technology in Disaster Medicine

Duan Yu-yu 1
Zhang Jia-yao 2
Xie Mao 2
Feng Xiao-bo 2
Xu Song 2
Ye Zhe-wei 2
1 grid.257143.6 0000 0004 1772 1285 The Clinical School of Chinese Medicine, Hubei University of Chinese Medicine, Wuhan, 430065 China
2 grid.33199.31 0000 0004 0368 7223 Department of Orthopaedics, Union Hospital, Tongji Medical College, Huazhong University of Science and Technology, Wuhan, 430022 China
Electronic publication date: 2021 Jan 11
Print publication date: 2020
Volume: 40
Issue: 6
First page: 1205
Last page: 1205
Copyright: © The Author(s) 2020
License: This article is licensed under a Creative Commons Attribution 4.0 International License https://creativecommons.org/licenses/by/4.0/), which permits use, sharing, adaptation, distribution and reproduction in any medium or format, as long as you give appropriate credit to the original author(s) and the source, provide a link to the Creative Commons licence, and indicate if changes were made. The images or other third party material in this article are included in the article’s Creative Commons licence, unless indicated otherwise in a credit line to the material. If material is not included in the article’s Creative Commons licence and your intended use is not permitted by statutory regulation or exceeds the permitted use, you will need to obtain permission directly from the copyright holder. To view a copy of this licence, visit http://creativecommons.org/licenses/by/4.0/.
License URL: https://creativecommons.org/licenses/by/4.0/

Erratum for: Application of Virtual Reality Technology in Disaster Medicine. 39 5 14 10 2019 690 693 Curr Med Sci 10.1007/s11596-019-2093-4 31612384
issue-copyright-statement: © Huazhong University of Science and Technology 2020

==============================
The original article can be found online at 10.1007/s11596-019-2093-4

